# Mutations Enabling Displacement of Tryptophan by 4-Fluorotryptophan as a Canonical Amino Acid of the Genetic Code

**DOI:** 10.1093/gbe/evu044

**Published:** 2014-02-25

**Authors:** Allen Chi-Shing Yu, Aldrin Kay-Yuen Yim, Wai-Kin Mat, Amy Hin-Yan Tong, Si Lok, Hong Xue, Stephen Kwok-Wing Tsui, J. Tze-Fei Wong, Ting-Fung Chan

**Affiliations:** ^1^School of Life Sciences, The Chinese University of Hong Kong, Hong Kong SAR, China; ^2^Hong Kong Bioinformatics Centre, The Chinese University of Hong Kong, Hong Kong SAR, China; ^3^Division of Life Science and Applied Genomics Center, The Hong Kong University of Science and Technology, Hong Kong SAR, China; ^4^Faculty of Medicine, The Chinese University of Hong Kong, Hong Kong SAR, China; ^5^School of Biomedical Sciences, The Chinese University of Hong Kong, Hong Kong SAR, China

**Keywords:** genetic code evolution, unnatural amino acids, amino acid analogs, *Bacillus subtilis*, fluorotryptophan

## Abstract

The 20 canonical amino acids of the genetic code have been invariant over 3 billion years of biological evolution. Although various aminoacyl-tRNA synthetases can charge their cognate tRNAs with amino acid analogs, there has been no known displacement of any canonical amino acid from the code. Experimental departure from this universal protein alphabet comprising the canonical amino acids was first achieved in the mutants of the *Bacillus subtilis* QB928 strain, which after serial selection and mutagenesis led to the HR23 strain that could use 4-fluorotryptophan (4FTrp) but not canonical tryptophan (Trp) for propagation. To gain insight into this displacement of Trp from the genetic code by 4FTrp, genome sequencing was performed on LC33 (a precursor strain of HR23), HR23, and TR7 (a revertant of HR23 that regained the capacity to propagate on Trp). Compared with QB928, the negative regulator *mtrB* of Trp transport was found to be knocked out in LC33, HR23, and TR7, and sigma factor *sigB* was mutated in HR23 and TR7. Moreover, *rpoBC* encoding RNA polymerase subunits were mutated in three independent isolates of TR7 relative to HR23. Increased expression of *sigB* was also observed in HR23 and in TR7 growing under 4FTrp. These findings indicated that stabilization of the genetic code can be provided by just a small number of analog-sensitive proteins, forming an oligogenic barrier that safeguards the canonical amino acids throughout biological evolution.

## Introduction

Although different organisms and organelles display minor variations with respect to the allocation of the 64 codons to the 20 amino acids (Knight et al. 2001; Silva et al. 2007), only the ensemble of 20 canonical amino acids are incorporated during translation among all known organisms. This encoded 20-amino-acid ensemble has remained immutable throughout 3 billion years of biological evolution as indicated by its universal adoption by all extant organisms. Despite the fact that amino acid analogs exist in nature, to date there is no evidence indicating that any one of the canonical amino acids has ever been completely replaced by its analog in the genetic code of an organism. However, departure from such conservation was first demonstrated experimentally by the stable mutants of parental *Bacillus subtilis* QB928 that were selected to propagate only on 4-fluorotryptophan (4FTrp), an indole-containing analog of tryptophan (Trp, Wong 1983; Mat et al. 2010). In fact, for the mutant HR23 strain, the supplanted Trp acted as an inhibitory analog and caused the formation of an inhibition zone on agar gel surrounding a well-containing Trp (Mat et al. 2010). Stable mutants of QB928 that propagate successfully on 5-fuoroTrp (5FTrp) and 6-fluoroTrp (6FTrp) have also been obtained even though 4FTrp, 5FTrp, and 6FTrp are potent growth inhibitors of the parental QB928 strain. In each instance, the growth of the organism on the analog has been confirmed by protein analysis based on fluorine-19 nuclear magnetic resonance (^19^F-NMR) and high-performance liquid chromatography (HPLC) (Mat et al. 2010). 4FTrp, 5FTrp, and 6FTrp are toxic indole-containing analogs of Trp (Browne et al. 1970; Pratt and Ho 1975), and they can be specifically charged by *B. subtilis* Trp-tRNA synthetase to tRNA^Trp^ with reduced efficiency (Xu et al. 1989).

The mutant strains of QB928 showed that the genetic code is not an immutable construct. Instead, the invariance of its canonical amino acids during the past 3 billion years has resulted from powerful mechanisms rigorously maintaining the stability of the present day code. Although the precise mechanisms remain to be delineated, the observation that Trp can be replaced or even displaced by an analog through a small number of mutational steps suggests that oligogenic barriers comprising a limited number of proteins are involved in the obligatory usage of the canonical amino acids. Possibly, the global incorporation of an amino acid analog during translation may result in loss of protein function, reduced overall fitness, and inhibition of cell propagation. Therefore, even as benign, an analog as hydroxy-Pro has failed to supplant Pro in any known biological lineage at the level of translation. Instead, in circumstances where proteins with modified side chains are required, either posttranslational modifications or encoding of selenocysteine (Berry et al. 1991) and pyrrolysine (Krzycki 2004; Polycarpo et al. 2004) by stop codons have been recruited to introduce novel side chains into the proteins.

To gain insight into the validity of these plausible oligogenic barriers, we have set out in this study to characterize the genomic and transcriptomic changes occurring in five *B. subtilis* genetic code mutants of parental QB928 with varying capabilities to propagate on Trp and 4FTrp by high-throughput DNA sequencing and RNA sequencing.

## Materials and Methods

### Growth Curve Analysis

**Fig. 1.—:**
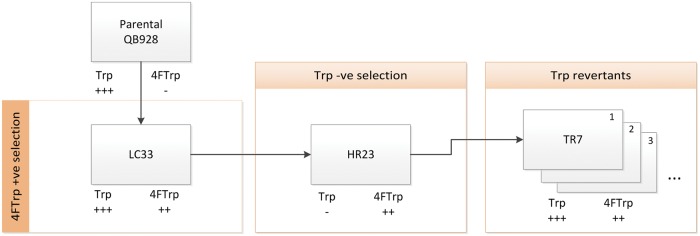
Relationships of sequenced strains. Strain selection conditions are indicated, and differential propagations of each strain on Trp/4FTrp are represented by “+” or “−,” where higher number of “+” symbols represents faster propagation, and "−" represents the inability to propagate.

**Fig. 2.—:**
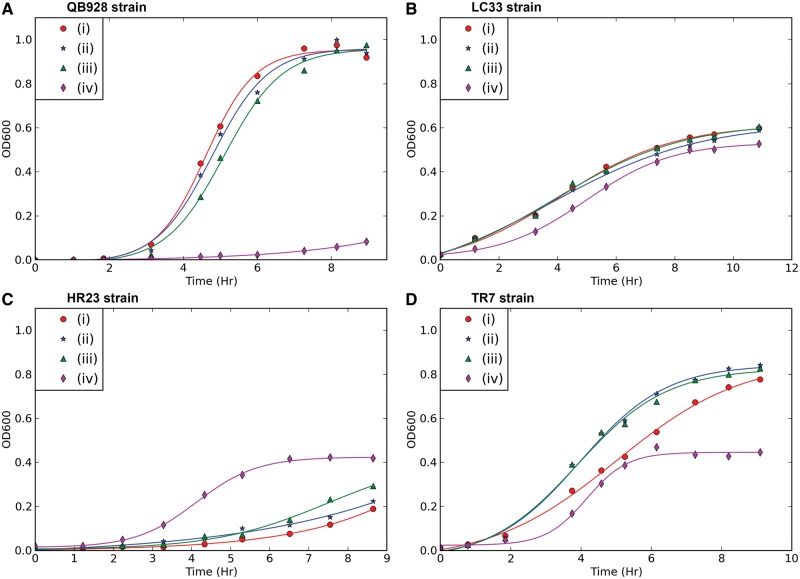
Short-term growth curves of (*A*) QB928, (*B*) LC33, (*C*) HR23, and (*D*) revertant TR7-1. The four medium G-based growth media employed differed in Trp/4FTrp contents: (i) supplemented with Trp only, (ii) Trp and 4FTrp in 2:1 ratio, (iii) Trp and 4FTrp in 1:2 ratio, and (iv) 4FTrp only.

The isolation of the mutant strains of Trp-auxotrophic *B. subtilis* QB928 (*aroI906 purB33 dal trpC_2_*) in figure 1 was described previously (Mat et al. 2010). *Bacillus subtilis* strains were first grown on 20 ml 1.6% agar plates containing Medium G (MG) (Wong 1983) and 5 µg/ml Trp or 4FTrp at 37 °C. For the experiments in figure 2, QB928, LC33, and TR7-1 were each grown up overnight at 37 °C, shaken at 200 rpm, and again to mid-log phase in MG supplemented with 5 µg/ml Trp, whereupon the cells were washed and allowed to resume growth under conditions 1) containing Trp only, 2) Trp and 4FTrp in 2:1 ratio, 3) Trp and 4FTrp in 1:2 ratio, and 4) containing 4FTrp only, with Trp and 4FTrp in each instance totaling 5 µg/ml. HR23, however, was grown up overnight and again to mid-log in Medium G supplemented with 5 µg/ml 4FTrp before washing and resumption of growth under conditions 1)–4). Optical density was measured on Thermo Biomate 3S at 600 nm for triplicate cultures, and the averaged measurements were fitted to Richard’s curve (Richards 1959) by the least squares method in Scipy package.

### High-Throughput DNA Sequencing

DNA was isolated from single-colony overnight cultures of each strain using DNAzol (Chomczynski et al. 1997). High-throughput DNA sequencing was performed on Illumina GA IIx 75 bp paired-end platform (∼200 bp insert size) at BGI-Shenzhen (BGI) and the Genome Research Center (GRC), University of Hong Kong. Sequence libraries were prepared using Illumina kits with standard protocol. Raw sequencing reads were deposited to National Center for Biotechnology Information (NCBI) Sequence Read Archive (accession ID: SRA057077). The reads from each strain were mapped to the reference QB928 genome (Yu et al. 2012) using SHRiMP 2.1.1 (David et al. 2011) (-p opp-in -E -Q –single-best-mapping –half-paired). Gene annotations were originated from *B. subtilis str.168* (Barbe et al. 2009), with refinements described previously (Yu et al. 2012). Sequencing depths of the different genomes are shown in supplementary table S1, Supplementary Material online.

### Variant Calling, Annotation, and Confirmation

Mutations were called against reference QB928 genome (GenBank: NC_018520.1) using SAMTools mpileup pipeline (Li et al. 2009) and filtered according to the criterion of >4 reads covered and mutant allele frequency >0.6. Mutations were confirmed by direct visual inspection of the sequence alignment and by polymerase chain reaction followed with Sanger sequencing. In-house scripts were used to annotate amino acid changes with respect to QB928 protein sequences and the affected Pfam protein domains (Punta et al. 2012) resulting from the mutations. Mutations in intergenic regions were further annotated using DBTBS (Sierro et al. 2008) to determine whether they were located within promoters, transcription factor binding sites, or terminators. To estimate the likelihood that a mutation impacted the protein functionally, predictions were made based on PROVEAN (Choi et al. 2012). Functional impact (FI) scores ≤ −2.0 are considered as deleterious, whereas FI scores > −2.0 are considered as neutral.

### RNA Sequencing

To study changes in gene expression level in Trp and 4FTrp, we have performed strand-specific RNA sequencing on all possible growth conditions of HR23, TR7, together with the parental QB928. Strand-specific paired-end library construction method was described in detail previously (Parkhomchuk et al. 2009). RNA sequencing was done on the Illumina GA IIx 75 bp paired-end platform (∼200 bp insert size) at the GRC, the University of Hong Kong.

Read counts of transcripts and fragments per kilobase of transcript per million mapped reads were analyzed using Cufflinks 2.1.1(Trapnell et al. 2010; Roberts et al. 2011), followed by differential gene expression analysis using cuffdiff. Biocyc version 17 (Caspi et al. 2008), and Pathway Tools 17 (Karp et al. 2010) were used to look for direct regulates of sigma factors.

## Results

### Growth of Parental Strain and Mutants on Trp and 4FTrp

The isolations and phenotypes of genetic code mutants of *B. subtilis* QB928 with altered indole-amino acid requirements for propagation are shown in figure 1. When monitored for cell propagation based on colony formation or ^33^P-phosphate incorporation on agar, the QB928 parental strain propagated well on Trp but not 4FTrp. QB928 gave rise serially to the LC33 mutant, which propagated on both Trp and 4FTrp, albeit slower on 4FTrp than on Trp. Further mutagenesis of LC33 gave rise to HR23, which propagated well on 4FTrp but not on Trp. However, isolates of the revertant TR7 strain derived from HR23 regained the capacity to propagate on either Trp or 4FTrp, in fact propagating faster on Trp than on 4FTrp (Mat et al. 2010).

In figure 2, parental QB928 and the LC33, HR23, and TR7-1 mutants were grown in suspension in four growth media: 1) containing Trp only; 2) Trp and 4FTrp in 2:1 ratio; 3) Trp and 4FTrp in 1:2 ratio; and 4) containing 4FTrp only. The short-term growth curves observed for these strains in media 1)–4) were consistent with the relative capacities of Trp and 4FTrp to support the long-term propagation of these strains on agar (Mat et al. 2010). Thus, QB928 underwent much slower short-term biomass increase on 4FTrp in 4) compared with 1)–3) in the presence of Trp. On the other hand, LC33 underwent moderate biomass increase in all growth medium 1)–4), the rate of increase on 4FTrp being slower than that on Trp. For HR23, although there was visible short-term biomass increases in 1)–3) in the presence of Trp, these increases were smaller than that obtained in 4) in the presence of 4FTrp alone, thus showing the action of Trp as an inhibitory analog on this strain. Finally, for revertant TR7-1, the biomass increases attained in 1)–3) in the presence of Trp surpassed that in 4) in the presence of 4FTrp alone. Therefore, the genetic changes in mutants LC33, HR23, and TR7 affected both long-term cell propagation and short-term biomass increase.

### Mutations Occurring between QB928 and TR7

The mutations associated in the sequential transitions from QB928 to LC33, HR23, and finally TR7 are presented in tables 1–3. As categorized in table 4, the genomic sequence of LC33 displayed, in comparison to the QB928 genomic sequence (Yu et al. 2012), 5 indels and 35 base substitutions, the latter including 9 missense mutations, 2 premature terminations, 12 synonymous mutations, and 12 mutations in intergenic regions. Unlike all other mutant isolation steps in figure 1, which were conducted without chemical mutagenesis, N-methyl-N’-nitro-N-nitrosoguanidine mutagenesis was performed in mutating LC33. Therefore, not surprisingly, a much larger number of mutations were found in HR23 compared with LC33, with 9 indels and 67 base substitutions, the latter including 26 missense mutations, 6 premature terminations, and 21 synonymous mutations. Revertants TR7-1 and TR7-2, derived from HR23, each displayed only two missense mutations and one synonymous mutation; TR7-1 also showed three indels.

**Table 1:** Nonsilent Mutations Appearing in LC33 Relative to QB928^a^

COG	Gene	Product	Type	Mutation	LC33	HR23	TR7-1	TR7-2	FI
C	*nhaC*	Na+/H+ antiporter	MISSENSE	Asp403Gly	+	+	+	+	3.95
E	*hom*	Homoserine dehydrogenase	MISSENSE	Ala191Val	+	+	+	+	−3.62
E	*mtrB*	Tryptophan operon RNA-binding attenuation protein (TRAP)	NONSENSE	Gln47Stop	+	+	+	+	NA
K	*yesS*	Transcriptional regulator (AraC/XylS family)	MISSENSE	Thr285Pro	+	+	+	+	4.27
K	*ytlI*	Transcriptional regulator (LysR family)	MISSENSE	Met6Ile	+	+	+	+	1.13
P	*znuB*	High affinity Zn(II) ABC transporter (permease)	FRAMESHIFT INSERTION	T(514)TTC	+	+	+	+	NA
Q	*srfAA*	Surfactin synthetase	MISSENSE	Val2743Phe	+	+	+	+	6.78
T	*kinA*	Sporulation-specific ATP-dependent protein histidine kinase	NONSENSE	Leu21Stop	+	+	+	+	NA
T	*lytS*	Two-component sensor histidine kinase [LytT]	MISSENSE	Ala507Val	+	+	+	+	−1.57
T	*prpE*	Phosphorylated protein phosphatase and diadenosine-polyphosphate hydrolase	MISSENSE	Tyr29Asn	+	+	+	+	3.59
T	*resE*	Two-component sensor histidine kinase	MISSENSE	Lys252Asn	+	+	+	+	−5.00
T	*resE*	Two-component sensor histidine kinase	MISSENSE	Gln250Pro	+	—	—	—	−6.00

Note.—NA indicates FI was not analyzed. For insertions, the inserted base is denoted in parentheses.

^a^FI was determined using PROVEAN (Choi et al. 2012). FI scores ≤ −2 are considered as deleterious, whereas FI scores > −2 are considered as neutral.

**Table 2:** Nonsilent Mutations Appearing in HR23 Relative to LC33

COG	Gene	Product	Type	Mutation	HR23	TR7-1	TR7-2	FI
C	*atpD*	ATP synthase (subunit beta, component F1)	MISSENSE	Ile175Leu	+	+	+	−2.00
C	*cydA*	Cytochrome bd ubiquinol oxidase (subunit I)	MISSENSE	Ser27Leu	+	+	+	0.12
C	*ctaO*	Protoheme IX farnesyltransferase (heme O synthase)	MISSENSE	Leu3Pro	+	+	+	−0.05
C	*ndh*	NADH dehydrogenase	MISSENSE	Thr255Ile	+	+	+	−4.78
E	*murAA*	UDP-N-acetylglucosamine 1-carboxyvinyltransferase	MISSENSE	Met92Leu	+	+	+	−3.00
E	*trpE*	Anthranilate synthase	MISSENSE	Ala466Pro	+	+	+	−4.90
E	*yxiO*	Putative efflux transporter	MISSENSE	Ala66Thr	+	+	+	−2.53
F	*hprT*	Hypoxanthine-guanine phosphoribosyltransferase	MISSENSE	Ala50Thr	+	+	+	−0.86
J	*ileS*	Isoleucyl-tRNA synthetase	MISSENSE	Glu814Gly	+	+	+	−6.88
J	*infB*	Initiation factor IF-2	NONSENSE	Arg87Stop	+	+	+	NA
J	*infB*	Initiation factor IF-2	NONSENSE	Lys85Stop	+	+	+	NA
J	*yugI*	Putative RNA degradation protein; putative phosphorylase or nucleotidyl transferase	FRAMESHIFT INSERTION	T(251)TCAGGCGCA	+	+	+	NA
K	*sigB*	RNAP sigma-37 factor (sigma(B))	MISSENSE	Met64Leu	+	+	+	−2.36
K	*sigI*	RNAP sigma-I factor	NONSENSE	Glu23Stop	+	+	+	NA
K	*ydeB*	Putative transcriptional regulator	MISSENSE	Met43Ile	+	+	+	−3.13
L	*recA*	Multifunctional SOS repair factor	MISSENSE	Ile138Val	+	+	+	−0.41
L	*ypsC*	Putative methylase with RNA interaction domain	MISSENSE	Glu297Asp	+	+	+	−1.33
M	*lytH*	Sporulation-specific L-Ala-D-Glu endopeptidase	NONSENSE	Trp192Stop	+	+	+	NA
M	*ytkA*	Putative lipoprotein	MISSENSE	Ser110Tyr	+	+	+	−2.99
O	*clpP*	ATP-dependent Clp protease proteolytic subunit	MISSENSE	Gly159Val	+	+	+	−8.80
P	*perR*	Transcriptional regulator (Fur family)	MISSENSE	Thr21Ser	+	+	+	−3.96
P	*yfiY*	Putative iron(III) dicitrate transporter binding lipoprotein	MISSENSE	Ser190Tyr	+	+	+	−2.46
R	*yabN*	Putative fusion methylase and nucleotide pyrophosphohydrolase	MISSENSE	Asn51Ile	+	+	+	−1.40
R	*ybdN*	Putative phage protein	MISSENSE	Ser222Arg	+	+	+	−1.81
R	*ydiB*	Putative ATPase or kinase UPF0079	MISSENSE	Glu34Asp	+	+	+	−2.77
R	*yocS*	Putative sodium-dependent transporter	MISSENSE	Met136Ile	+	+	+	−2.68
R	*ytkL*	Putative metal-dependent hydrolase	FRAMESHIFT INSERTION	G(35)GT	+	+	+	NA
S	*yheB*	Conserved hypothetical protein	MISSENSE	Leu358Phe	+	+	+	−3.86
S	*yndB*	Conserved hypothetical protein	MISSENSE	Ser75Phe	+	+	+	−4.73
S	*yomD*	Conserved hypothetical protein; phage SPbeta	NONSENSE	Leu10Stop	+	+	+	NA
V	*bceB*	ABC transporter (permease)	MISSENSE	Met583Ile	+	+	+	0.08
V	*sdpB*	Exporter of killing factor SpbC	MISSENSE	Glu55Lys	+	+	+	−0.98
V	*vmlR*	ATP-binding cassette efflux transporter	NONSENSE	Gly117Stop	+	+	+	NA
V	*vmlR*	ATP-binding cassette efflux transporter	MISSENSE	Glu120Lys	+	+	+	0.30

**Table 3:** Nonsilent Mutations Appearing in Different Isolates of TR7 Relative to HR23

COG	Gene	Product	Mutation Type	Mutation	TR7-1	TR7-2	TR7-3	FI
K	*rpoB*	RNAP (β subunit)	MISSENSE	Glu433Lys	+	—	—	−4.00
K	*rpoC*	RNAP (β′ subunit)	MISSENSE	Ile280Thr	—	+	—	−5.00
K	*rpoC*	RNAP (β′ subunit)	MISSENSE	Pro277His	—	—	+	−9.00
T	*resD*	Two-component response regulator	FRAMESHIFT INSERTION	T(245)TTA	+	—	—	NA
T	*resD*	Two-component response regulator	MISSENSE	Arg201Gly	—	+	—	−6.99

**Table 4:** Overview of Mutations in Different Mutant Strains^a^

	LC33	HR23	TR7-1	TR7-2
Synonymous	12	21	1	1
Missense	9	26	2	2
Premature termination	2	6	0	0
Noncoding	12	14	0	0
Total indels	5	9	3	0
Total substitutions	35	67	3	3
Total mutations	40	76	6	3

^a^Mutations in LC33 were scored with respect to QB928; ones in HR23 scored with respect to LC33; and ones in TR7-1 or TR7-2 scored with respect to HR23.

### Nonsynonymous Mutation Rate Is Not Correlated to Trp-Residue Density

**Fig. 3.—:**
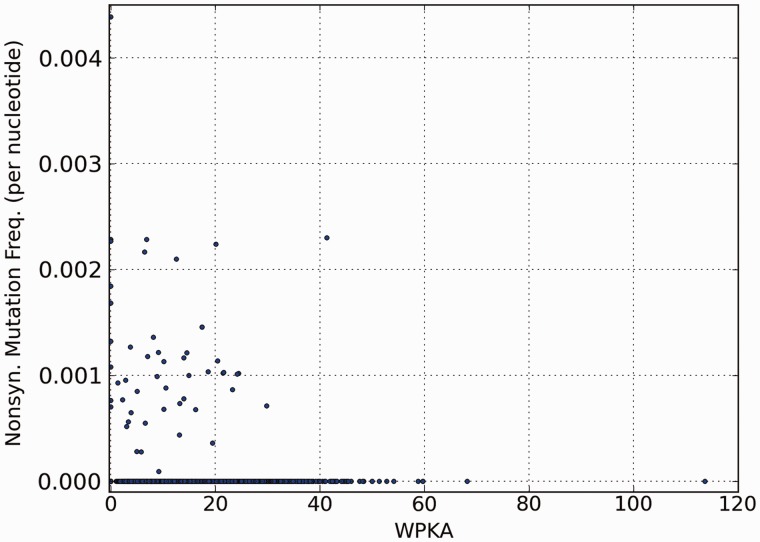
Variation of nonsynonymous point mutation frequency with tryptophan content. WPKA represents tryptophan per kilo amino acids. According to Spearman correlation test, there is no significant correlation between nonsynonymous point mutation frequency and WPKA, as indicated by ρ = 0.01 and *P* value = 0.55.

As 4FTrp was toxic to the parental QB928 strain, the selection pressure on proteins rich in Trp residues might be higher than Trp-deficient proteins, thereby accumulating a larger number of nonsynonymous mutations. Contrary to this expectation, figure 3 shows no significant correlation between the nonsynonymous mutation rate for genes found in the sequenced genomes and the Trp-residue density of the mutated proteins measured by WPKA, namely number of Trp residues per kilo amino acids, with Spearman correlation = 0.01 and *P* value = 0.55. Another potential mechanism for the cells to adapt to 4FTrp-supported growth might entail reduction of Trp residues in the proteome through mutations. However, table 5 shows no significant difference between the nonsilent mutation rate of Trp residues and that of non-Trp residues in the sequenced genomes, yielding *P* value = 0.45 based on Fisher’s exact test, indicating the absence of any significant difference between the two mutation rates. Therefore, it is unlikely that the selection pressure due to 4FTrp incorporation was exerted globally throughout the proteome.

**Table 5:** Nonsilent Mutations at Trp versus Non-Trp Residues in the Combined Genomic Sequences of LC33, HR23, TR7-1, and TR7-2

Original Residue	Mutated Residue	Unmutated Residue	Row Total
Trp	1	12,464	12,465
Non-Trp	57	1,195,138	1,195,195
Column total	58	1,207,602	1,207,660

Note.—Based on Fisher’s exact test, no significant difference between the two mutation rates were observed, as indicated by *P* value = 0.45.

### Genic Changes Enabling Cell Propagation on 4FTrp

When parental QB928 was mutated to LC33, the ability to propagate on 4FTrp was greatly enhanced (Wong 1983). In table 1, the conversion of QB928 to LC33 was accompanied by 12 missense, nonsense, or frameshift insertion mutations, residing in 11 different genes from six different Clusters of Orthologous Groups (COGs) (Tatusov et al. 2003): C—energy production and conversion; E—amino acid metabolism and transport; K—transcription; P—inorganic ion transport and metabolism; Q—secondary metabolites biosynthesis, transport and catabolism; and T—signal transduction mechanisms. Nonsense and frameshift mutations could often result in a high FI on the protein. For any missense mutation, its FI was assessed by PROVEAN (Choi et al. 2012), which measured the impact of mutations in amino acid sequences using an alignment-based score with the assumption that variations in evolutionary conserved regions would be more deleterious. A score threshold of ≤−2.0 was used to classify a variant as having potential deleterious effect. Altogether six of the mutations shown in table 1 were potentially deleterious, being a nonsense mutation, a frameshift insertion mutation, or a missense mutation with FI ≤ −2.0.

### Genic Changes Leading to Failure of Trp to Support Propagation

LC33 could propagate on Trp, but HR23 could not. In fact, Trp inhibited the growth of HR23, causing an inhibition zone on a lawn of HR23 cells on 4FTrp-supplemented agar (Mat et al. 2010). Table 2 shows the 34 missense, nonsense, or frameshift insertion mutations in 32 genes found in the genome of HR23 relative to that of LC33. They arose in 12 different COGs: C, E, K, and P; F—nucleotide transport and metabolism; J—translation, ribosomal structure, and biogenesis; L—replication, recombination, and repair; M—cell wall/membrane/envelope biogenesis; O—posttranslational modification, protein turnover, chaperones; R—general function prediction; S—function unknown; and V—defense mechanisms. Therefore, the number of both mutated genes and COGs involved were more numerous in the conversion of LC33 to HR23 compared with the conversion of QB928 to LC33. Of these mutations, 24 of them were nonsense, frameshift insertion, or missense mutations with FI ≤ −2.0 and therefore potentially causing a significant FI.

### Genetic Reversion of HR23 Cells to Support Propagation on Trp

When HR23 cells were grown as a lawn on 4FTrp-agar, the inhibition zone surrounding a well-containing Trp provided means to isolate revertants of HR23 that regained the ability to propagate on Trp, appearing as colonies inside the inhibition zone. On this basis, revertant TR7 was isolated, which propagated on Trp and 4FTrp but not on 6FTrp or 5FTrp (Mat et al. 2010). In this study, an additional revertant TR7-2 was isolated by the same procedure, and the original TR7 strain was renamed TR7-1. Table 3 presents the mutations found in the genomes of revertants TR7-1 and TR7-2 relative to parental HR23. In both of these TR7 isolates, mutations were found in the β or β′ subunits of the RNA polymerase (RNAP), encoded by *rpoB* or *rpoC,* respectively, and in *resD* which together with its downstream *resE* gene encode the two-component signal transduction system ResDE (Nakano et al. 1996). Because both TR7-1 and TR7-2 displayed mutations in the *rpo* and *resD* genes, the *rpoB, rpoC*, *resD,* and *resE* genes of an additional isolate TR7-3 were sequenced by Sanger DNA sequencing. Thereupon, TR7-3 was found to harbor a mutation in *rpoC* different from that found in TR7-2 but no mutation in either *rpoB* or *resDE.*

### Expression Level of Genes Related to Trp Metabolism

**Fig. 4.—:**
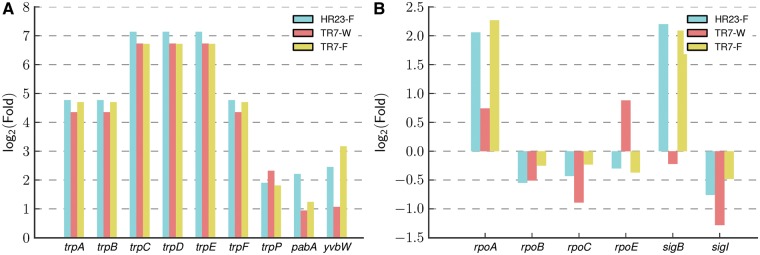
Expression fold change of (*A*) genes in Trp biosynthetic pathway and (*B*) RNAP subunits and mutated sigma factors. Log_2_-transformed expression fold changes with respect to QB928-W are shown.

Although none of the Trp-auxotrophic QB928 and its descendant strains in figure 1 could synthesize Trp or 4FTrp (Mat et al. 2010), it would be of interest to examine the regulation of genes related to Trp metabolism under conditions of Trp or 4FTrp utilization by the cells. Accordingly, transcription analysis was performed by means of RNA sequencing on QB928 growing on Trp (QB928-W cells), HR23 growing on 4FTrp (HR23-F), and TR7-1 growing on either Trp (TR7-W) or 4FTrp (TR7-F). As shown in figure 4 and supplementary table S2, Supplementary Material online, the genes related to the Trp biosynthesis pathway were overexpressed in the HR23-F, TR7-W, and TR7-F relative to QB928-W cells. In particular, the *trpEDCFBA* genes in the Trp operon were up-regulated >4.87 log_2_-fold. The two genes that were responsible for Trp transport, *yvbW* and *trpP*, were also up-regulated 1.18–3.61 log_2_-fold in HR23-F, TR7-W, and TR7-F. These results suggest that the expressions of genes related to Trp metabolism were widely up-regulated in feedback response to growth limitation by availability of Trp or 4FTrp inside the HR23-F, TR7-W, and TR7-F cells.

### Expressions of RNAP and Sigma Factors

The parental HR23 strain, which could not propagate on Trp, and its three independent TR7 revertants, which regained the ability to propagate on Trp, each contained two mutated sigma factors, namely SigB Met64Leu and SigI Glu23Stop (table 2). The finding that all three TR7 isolates contained a nonsilent mutation in RNAP (table 3) was therefore noteworthy. Because *B. subtilis* sigma factors control the specificity of RNAP toward its cognate promoters (Haldenwang 1995), the possibility has to be considered that the mutated RNAP and the mutated sigma factors might interact to determine the rejection or restoration of Trp as a propagation-supporting amino acid.

Accordingly, the mRNA levels of the mutated *rpoB*, *rpoC*, *sigB,* and *sigI* were measured along with the nonmutated *rpoA* and *rpoE* (fig. 4, supplementary table S3, Supplementary Material online). For *rpoB, rpoC, rpoE,* and *sigI*, only small changes in expression level were observed, where the absolute log_2_-fold changes with respect to QB928-W were <1.3. In contrast, the expression levels of *rpoA* and *sigB* were highly up-regulated by >2 log_2_-fold in HR23-F and TR7-F but not in TR7-W conditions (fig. 4, supplementary table S3, Supplementary Material online).

## Discussion

### Postulate of Oligogenic Barriers against Amino Acid Analogs

In view of the rigorous conservation of the 20 canonical amino acids encoded by the genetic code, it was surprising that only several rounds of mutations sufficed to enable *B. subtilis* QB928 cells to propagate on the normally inhibitory Trp analogs 4FTrp, 5FTrp, and 6FTrp (Mat et al. 2010). The presence of nonsynonymous mutations among proteins also was not correlated with either the frequency of Trp residues in a protein (fig. 3) or enriched at Trp residues (table 5). These findings suggest that the main effects of 4FTrp incorporation were localized at a small number of FTrp-sensitive proteins containing critical Trp residue(s) that underwent gross dysfunction upon substitution of Trp with FTrp, whereas the majority of Trp-containing proteins in the *B. subtilis* proteome functioned adequately with either Trp or 4FTrp (and likewise 5FTrp or 6FTrp). Accordingly, there might exist a strong oligogenic barrier, comprising a relatively small number of genes, that would ensure the maintenance of cell viability through usage of only the canonical amino acids, thereby blocking cell propagation on analogs such as the FTrps (Mat et al. 2010).

Based on this oligogenic barrier postulate, when the 4FTrp-sensitive proteins were mutated in LC33 so that the dysfunctions caused by 4FTrp incorporation were moderated, 4FTrp would become a propagation-supporting amino acid. Conversely, when one or more proteins in the LC33 proteome mutated to become sensitive to Trp, the resultant HR23 cells could propagate on 4FTrp but no longer on Trp, and Trp became thereupon an inhibitory analog of 4FTrp. In the TR7 revertants, the Trp-induced dysfunction was overcome by further mutations, and propagation on Trp was once again allowed. During biological evolution, such oligogenic barriers would channel the acquisition of beneficial novel amino acid side chains toward posttranslational modifications instead of unending expansion of the amino acid alphabet encoded by the canonical genetic code.

Clearly, the only way to test the postulated existence of oligogenic barriers as a key factor for the hitherto invariance of the canonical genetic code was to conduct an actual count of the mutations required to open up the genetic code to functionally accept an analog such as 4FTrp. Whole-genome sequencing and the tabulation of genomic alterations in the course of the transition to 4FTrp utilization, followed by Trp rejection, thus represent an important first step to elucidate the underlying mechanisms determining the capability of Trp or 4FTrp to sustain cell viability and propagation.

Mutations listed in tables 1–3 inevitably contain passenger mutations that do not contribute to the oligogenic barrier significantly. In table 1, six of the mutations in LC33 were characterized with FI scores larger than −2.0 and therefore possibly passenger mutations because of their insignificant predicted impact. Besides, *hom* encodes homoserine dehydrogenase, which catalyzes the formation of homoserine as precursor to Thr, Ile, and Met. Because the Medium G employed in the growth and propagation of these cells was supplemented with Thr, Ile, and Met, the effects of the *hom* Ala191Val mutation might be limited. The remaining four mutated genes carrying five mutations out of a total of 4,034 protein coding genes in the genome readily met the description of an oligogenic barrier against the utilization of 4FTrp as an effective building block, the circumvention of which through mutations could serve to enhance cell propagation on 4FTrp.

HR23, derived from LC33 with the use of chemical mutagenesis during selection steps, lost the ability to propagate on Trp. In table 2, ten of the mutations in HR23 were characterized by an insignificant FI score of >−2.0 and thus might be passenger mutations. Although the mutation counts in table 1 readily validated the existence of an oligogenic barrier restricting the utility of 4FTrp for propagation of QB928, the larger number of 24 plausibly functional mutations in table 2 suggested either 1) LC33 had to undergo a larger number of mutations to reject Trp as an effective amino acid building block or 2) the 24 mutations with FI ≤ −2.0 might include some passenger mutations on proteins possibly nonessential to cell propagation.

As expected, mutations in genes related to Trp metabolism and stress response can be found, but we also observed numerous mutations in the transcription and translation machinery. Among the latter 24 mutations, the Glu814Gly mutation of IleRS was intriguing insofar that Ile activation was functionally removed from TrpRS activity. However, IleRS has a proofreading domain (Jakubowski 2012), and it would be of interest to determine whether this mutation could affect the defense of IleRS against inhibition or mischarging by 4FTrp, which is more hydrophobic than Trp with a ΔΔ*G* of 0.42 kcal/mol for transfer from n-octanol to water (Xu et al. 1989). The *ydiB* gene product, based on the latest annotation of *B. subtilis str.BSU168* genome (Belda et al. 2013), is responsible for the biosynthesis of the modified nucleoside threonylcarbamoyladensoine on tRNAs (Karst et al. 2009; Lauhon 2012) and could play an important role in the fine tuning of translation.

In contrast to the 24 plausibly functional mutations that accompanied the transition from LC33 to HR23 and the rejection of Trp as a canonical amino acid, the two nonsilent mutations displayed by each of the revertants TR7-1 and TR7-2 relative to HR23 (table 3) revealed that a maximum of two mutations sufficed to break down the barrier in HR23 against cell propagation on Trp, restoring to TR7-1 the capacity to propagate on Trp, in fact 40% faster than propagation on 4FTrp (Mat et al. 2010). The paucity of mutations displayed by TR7-1 and TR7-2 strongly supported the postulate of oligogeneic barriers as a key factor in rigorously preserving the composition of the canonical amino acid alphabet of the genetic code.

### Expression of Genes Related to Trp Transport and Biosynthesis

Previously, we observed that a Ser82Leu mutation on the *trpS* gene of *B. subtilis* for Trp-tRNA synthetase conferred resistance to growth inhibition by 5FTrp (Chow and Wong 1996). In contrast, although the transition of QB928 successively to LC33, HR23, and finally TR7 involved at each stage an alteration in the Trp/4FTrp propagation ratio, there was no mutation in *trpS* gene in tables 1, 2, or 3.

In contrast, the *mtrB* gene for the Trp operon RNA-binding attenuation protein (TRAP) underwent a Gln47Stop nonsense mutation near the N-terminus of the protein in LC33, which persisted in HR23, TR7-1, and TR7-2. TRAP is responsible for both transcription attenuation of Trp operon (Gollnick et al. 1990; Yanofsky 2007) and translational repression of *trpG, trpP,* and *ycbK* (Du et al. 1997; Sarsero et al. 2000; Yakhnin et al. 2004, 2006), and *mtrB* knockouts enhanced 5FTrp resistance (Gollnick et al. 1990). In *Escherichia coli,* a nonsense mutation in the aromatic amino acid regulon repressor *tyrR*, which is structurally and functionally similar to *mtrB*, also enhanced resistance of 4FTrp (Bacher and Ellington 2001). Thus, the Gln47Stop mutation in MtrB would help to explain the extensively elevated expression of the Trp transporter and Trp biosynthetic genes in HR23 growing on 4FTrp and TR7-1 growing on either Trp or 4FTrp. Notably, the elevated expression of Trp transporters could improve cell growth on Trp or 4FTrp, but the elevated expression of Trp biosynthetic genes would be futile on account of the *aroI906, trpC_2_* mutations rendering QB928, and all its derivative strains incapable of Trp biosynthesis.

### RNAP Mutations

HR23 could not propagate on Trp, but its TR7 revertants could do so. Upon sequencing, the genomes of the independent isolates TR7-1 and TR7-2 each yielded only two nonsilent mutations relative to the HR23 genome, namely Glu433Lys of RpoB and T(245)TTA of *resD* in TR7-1, and Ile280Thr of RpoC and Arg201Gly of ResD in TR7-2. In view of these results, the *rpoB*, *rpoC*, *resD,* and *resE* genes of a third independent revertant TR7-3 were also sequenced, which yielded a Pro277His mutation in RpoC but no mutation in either *rpoB* or *resD* (table 3). These findings indicated that *rpoBC* were candidate genes where appropriate mutations could determine the acceptability versus rejection of Trp as a canonical amino acid.

**Fig. 5.—:**
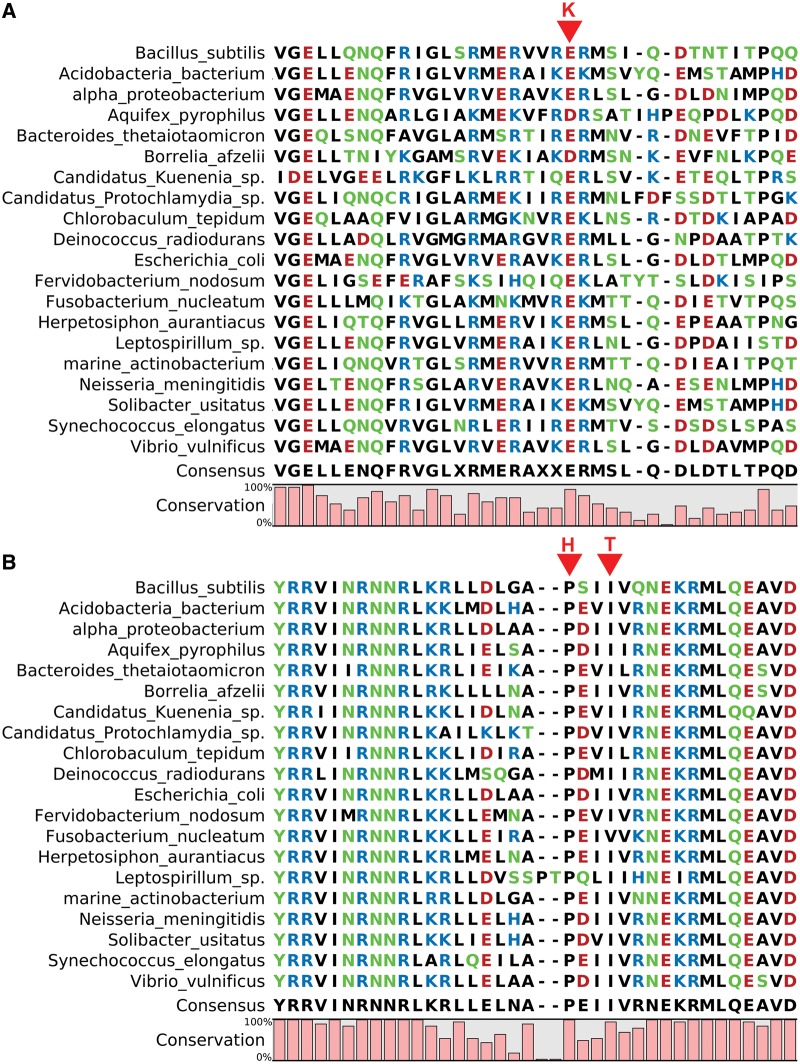
Multiple sequence alignment of bacterial RNAP β subunit (part *A*) and β′ subunit (part *B*). Portions of the full multiple sequence alignments of RpoB sequences from 960 bacterial strains and of RpoC sequences from 844 bacterial strains, identified by BlastP against NCBI nonredundant protein database, are shown. The mutated positions in strains TR7-1, TR7-3, and TR7-2 are marked by arrows with the respectively mutated residues K, H, and T indicated above the arrows. Because none of the mutated K, H, and T residues is found in the full multiple sequence alignments, their occurrences in the TR7 revertants are unique among reported bacterial RpoB and RpoC sequences.

**Fig. 6.—:**
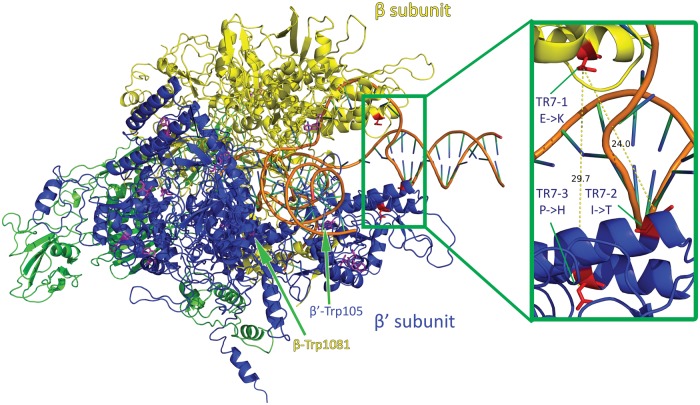
*Escherichia coli* RNAP transcription elongation complex (TEC) model (Opalka et al. 2010). RNAP β subunit is shown in yellow, β′ subunit in blue, Trp residues in magenta, and mutated residues in the independent isolates TR7-1, TR7-2, and TR7-3 in red within the green box. All three mutations are located at the outer claw-like region, one on the β subunit and two on the β′ subunit. The two universally conserved Trp residues, namely β-Trp1081 and β′-Trp105, are marked with green arrows. Distances in Ångström between the mutated K, H, and T residues are K-H 29.7 Å, K-T 24.0 Å, and H-T 13.7 Å, respectively.

Bacterial gene expression is dependent on the interaction between RNAP and different promoters, assisted in some cases by activator or repressor proteins (Haldenwang 1995). Different sigma factors can bind to the core RNAP subunits and modify the specificity of the α_2_ββ′ω RNAP (Cramer 2002; Lane and Darst 2010a, 2010b). Of the three missense RNAP mutations shown in table 3, one occurs on the β subunit and two on the β′ subunit. The β-Glu433, β′-Ile280, and β′-Pro277 residues involved are all strongly or totally conserved on the bacterial RNAP subunit sequences aligned in figure 5. Accordingly, the three mutations β-Glu433Lys, β′-Ile280Thr, and β′-Pro277His are characterized by the highly significant FI values of −4.00, −5.00, and −9.00, respectively. These three mutations were in fact not found on any of 960 RpoB and 844 RpoC sequences in the NCBI nonredundant protein database. The uniqueness of these mutations suggested that an extreme modification of the RNAP molecule was required to overcome the mutation(s) that effectively blocked cell propagation on Trp. Among the six Trp residues in the β subunit and seven Trp residues in the β′ subunit (marked in magenta in figure 6), two universally conserved Trp residues in bacterial RNAP, namely β-Trp1081 and β′-Trp105, are located in the lobe domain and clamp domain of RNAP, respectively (Cramer 2002; Lane and Darst 2010b). These two residues, and the other Trp residues in the two subunits, would merit investigation as potentially Trp-sensitive residues in RNAP in HR23.

In this regard, table 2 showed that the transition of LC33 to HR23 was accompanied by two mutations in sigma factors, which were carried over to both TR7-1 and TR7-2, namely Met64Leu in SigB and Glu23Stop in SigI. Accordingly, the modifications of RNAP exhibited by TR7-1, TR7-2, and TR7-3 could stem from the SigB Met64Leu and/or SigI Glu23Stop mutations. Supplementary table S3, Supplementary Material online, shows that, relative to QB928 cells growing on Trp, SigI level did not change greatly in HR23 cells growing on 4FTrp, or in TR7-1 cells growing on either Trp or 4FTrp. On the other hand, the expression level of SigB was increased by >2 log_2_-fold when HR23 or TR7-1 cells were grown on 4FTrp, suggesting that there could be some inadequacy in SigB performance inside these cells.

Consequently, the possibility exists that the SigB Met64Leu and/or SigI Glu23Stop mutations in HR23 was a major contributor to the failure of Trp to support cell propagation owing to inefficient interaction between α_2_ββ′ω RNAP and SigB and/or SigI, and this failure was remedied by the β-Glu433Lys, β′-Ile280Thr, or β′-Pro277His mutation displayed by TR7-1, TR7-2, and TR7-3, respectively. As shown in figure 6, this possibility is supported by the locations of these three mutations on the two sides of the conserved claw-like region on RNAP where sigma factor binds (Arthur et al. 2000; Murakami et al. 2002; Vassylyev et al. 2002). At their locations, each of these three mutations could be well positioned to rectify a malformation of the RNAP-SigB complex caused by the incorporation of Trp into RNAP or SigB or alleviate the dependence of essential RNAP functions on SigI, which would be knocked out by the Glu23Stop nonsense mutation near its N-terminus.

In conclusion, the invariance of the canonical amino acid alphabet throughout biological evolution has now been broken with the isolation of the *B. subtilis* QB928 genetic code mutants (fig. 1), where the ability of Trp to support growth can be reversibly displaced by 4FTrp. These findings have laid the foundation for synthetic lifeforms (Wong and Xue 2011). Moreover, the mutability of this canonical alphabet has since been confirmed by the development of additional approaches to introduce unnatural amino acids into proteomes, including the use of the thymidine auxotrophic *E. coli* R126L mutant where the analog azaleucine must be supplied to the cells to support growth in the absence of added thymidine (Lemeignan et al. 1993), the propagation of unColi strains and their phages on fluoroTrps (Bacher and Ellington 2001; Bacher et al. 2003, 2004), and the incorporation of a wide array of amino acid analogs via orthogonal pairs of tRNA and amino acyl-tRNA synthetase (Wang et al. 2001; Santoro et al. 2003; Xie and Schultz 2006). However, the mechanisms preserving the remarkable universality of the canonical amino acids encoded by the genetic code have remained largely unfathomed. In this regard, this study has provided strong evidence for oligogenic barriers encoding a small number of analog-sensitive proteins as a key factor in safeguarding the canonical alphabet throughout biological evolution. Notably, for Trp with its unique indole side chain, the number of metabolite analogs that could mount a challenge against its tenure as canonical amino acid through their activation by TrpRS and attachment to tRNA^Trp^ would be few. As a result, there was no need for the oligogenic barrier protecting the long lasting membership of Trp to include the TrpRS-encoding *trpS* gene, and tables 1 and 2 did not include any mutation of *trpS*. With a canonical amino acid such as Ile with a chemically less unique side chain, its cognate IleRS often has to be equipped with a proofreading domain to protect against mischarging of tRNA^Ile^ by Ile analogs. Therefore, the proofreading domain of IleRS may well be part of the oligogenic barrier for Ile that must be mutated to allow replacement or displacement of Ile from the genetic code by Ile analogs. Clearly, the oligogenic barrier will vary with both the canonical amino acid and the analog challenger.

In any event, a thorough understanding of the mechanisms upholding the canonical alphabets of proteins and nucleic acids in the living world, as well as other universally adopted building blocks and cofactors, will be required both to advance insights into the chemical parameters that shaped the living world and to enhance medicine and bioengineering through the design and introduction of additional building blocks.

## Supplementary Material

Supplementary tables S1–S3 are available at *Genome Biology and Evolution* online (http://www.gbe.oxfordjournals.org/).

Supplementary Data
