# Supplementary material for: Mutations Enabling Displacement of Tryptophan by 4-Fluorotryptophan as a Canonical Amino Acid of the Genetic Code
Source: Genome Biol Evol. 2014 Feb 25;6(3):629–41. doi: 10.1093/gbe/evu044 (PMC3971595; doi:10.1093/gbe/evu044)
Supplement: Supplementary Data [file supp_6_3_629__index.html]

Mutations Enabling Displacement of Tryptophan by 4-Fluorotryptophan as a Canonical Amino Acid of the Genetic Code — Mutations Enabling Displacement of Tryptophan by 4-Fluorotryptophan as a Canonical Amino Acid of the Genetic Code — Supplementary Data 

# Mutations Enabling Displacement of Tryptophan by 4-Fluorotryptophan as a Canonical Amino Acid of the Genetic Code

## Supplementary Data

files

**Files in this Data Supplement:**

- Supplementary Data - doc file
