# Supplementary material for: Mutations Enabling Displacement of Tryptophan by 4-Fluorotryptophan as a Canonical Amino Acid of the Genetic Code
Source: Genome Biol Evol. 2014 Feb 25;6(3):629–41. doi: 10.1093/gbe/evu044 (PMC3971595; doi:10.1093/gbe/evu044)
Supplement: Supplementary Data [file supp_evu044_BSU_manuscript_final_GBE_fork_20131118_supplementary.doc]

**Supplementary Table 1—Sequencing depths of different genomes**

| **Strain** | **Total Bases** | **Total Reads** | **Coverage** | **% Genes covered** |
| --- | --- | --- | --- | --- |
| LC33 | 351 Mb | 4744944 | 83.3 | 99.98 |
| HR23 | 345 Mb | 4663178 | 81.9 | 99.93 |
| TR7-1 | 1491 Mb | 21293965 | 353.6 | 99.98 |
| TR7-2 | 1232 Mb | 17603433 | 292.3 | 99.70 |

**Supplementary Table 2—Expression level of Trp transporters and genes in Trp biosynthetic pathway**

| **Gene** | **QB928-W exp U** | **HR23-F**  **exp U** | **TR7-W exp U** | **TR7-F**  **exp U** | **HR23-F**  **log_2_ Δ** | **TR7-W log_2_ Δ** | **TR7-F log_2_ Δ** |
| --- | --- | --- | --- | --- | --- | --- | --- |
| *trpA* | 7.75 | 12.51 | 12.09 | 12.44 | 4.77 | 4.35 | 4.70 |
| *trpB* | 7.75 | 12.51 | 12.09 | 12.44 | 4.77 | 4.35 | 4.70 |
| *trpC* | 5.45 | 12.56 | 12.15 | 12.13 | 7.14 | 6.73 | 6.72 |
| *trpD* | 5.45 | 12.56 | 12.15 | 12.13 | 7.14 | 6.73 | 6.72 |
| *trpE* | 5.45 | 12.56 | 12.15 | 12.13 | 7.14 | 6.73 | 6.72 |
| *trpF* | 7.75 | 12.51 | 12.09 | 12.44 | 4.77 | 4.35 | 4.70 |
| *trpP* | 10.53 | 12.43 | 12.85 | 12.35 | 1.90 | 2.32 | 1.81 |
| *pabA* | 9.45 | 11.66 | 10.39 | 10.69 | 2.21 | 0.94 | 1.24 |
| *yvbW* | 5.89 | 8.34 | 6.96 | 9.06 | 2.45 | 1.07 | 3.17 |

* List of genes in Trp biosynthetic pathway according to Bsubcyc .

exp U—Expression unit in log_2_ Fragment Per Kilobase of Transcript per Million mapped reads (log_2_FPKM)

log_2_ ∆—log_2_-expression fold change relative to QB928-W

**Supplementary Table 3—Expression level of *rpoABCE*, and *sigBI***.

| **Gene** | **QB928-W exp U** | **HR23-F**  **exp U** | **TR7-W exp U** | **TR7-F**  **exp U** | **HR23-F**  **log_2_ Δ** | **TR7-W log_2_ Δ** | **TR7-F log_2_ Δ** |
| --- | --- | --- | --- | --- | --- | --- | --- |
| *rpoA* | 10.66 | 12.27 | 11.23 | 12.50 | 2.06 | 0.74 | 2.27 |
| *rpoB* | 10.05 | 9.50 | 9.54 | 9.80 | -0.55 | -0.51 | -0.25 |
| *rpoC* | 11.00 | 10.58 | 10.11 | 10.77 | -0.43 | -0.89 | -0.23 |
| *rpoE* | 9.96 | 9.65 | 10.83 | 9.58 | -0.30 | 0.88 | -0.37 |
| *sigB* | 9.14 | 11.34 | 8.92 | 11.23 | 2.20 | -0.22 | 2.09 |
| *sigI* | 8.26 | 7.50 | 6.99 | 7.79 | -0.76 | -1.28 | -0.48 |
